# Assessment of the consistency of national-level policies and guidelines for malaria in pregnancy in five African countries

**DOI:** 10.1186/1475-2875-13-212

**Published:** 2014-06-03

**Authors:** Patricia P Gomez, Julie Gutman, Elaine Roman, Aimee Dickerson, Zandra H Andre, Susan Youll, Erin Eckert, Mary J Hamel

**Affiliations:** 1 Maternal and Child Health Integrated Program, Jhpiego, Baltimore, MD, USA; 2 Malaria Branch, Division of Parasitic Diseases and Malaria, Centers for Disease Control and Prevention, Atlanta, GA, USA; 3 President’s Malaria Initiative, Bureau for Global Health, U.S. Agency for International Development, Washington, DC, USA; 4 President’s Malaria Initiative, Malaria Branch, Division of Parasitic Diseases and Malaria, Centers for Disease Control and Prevention, Atlanta, GA, USA

**Keywords:** Malaria, Pregnancy, IPTp, SP, ITN, LLIN, Case management

## Abstract

**Background:**

At least 39 sub-Saharan African countries have policies on preventing malaria in pregnancy (MIP), including use of long-lasting insecticidal nets (LLINs), intermittent preventive treatment with sulphadoxine-pyrimethamine (IPTp-SP) and case management. However, coverage of LLINs and IPTp-SP remains below international targets in most countries. One factor contributing to low coverage may be that MIP policies typically are developed by national malaria control programmes (NMCPs), but are implemented through national reproductive health (RH) programmes.

**Methods:**

National-level MIP policies, guidelines, and training documents from NMCPs and RH programmes in Kenya, Mali, Mozambique, mainland Tanzania and Uganda were reviewed to assess whether they reflected WHO guidelines for prevention and treatment of MIP, and how consistent MIP content was across documents from the same country. Documents were compared for adherence to WHO guidance concerning IPTp-SP timing and dose, directly observed therapy, promotion and distribution of LLINs, linkages to HIV programmes and MIP case management.

**Results:**

The five countries reviewed had national documents promoting IPTp-SP, LLINs and MIP case management. WHO guidance from 2004 frequently was not reflected: four countries recommended the first dose of IPTp-SP at 20 weeks or later (instead of 16 weeks), and three countries restricted the first and second IPTp-SP doses to specific gestational weeks. Documents from four countries provided conflicting guidance on MIP prevention for HIV-positive women, and none provided complete guidance on management of uncomplicated and severe malaria during pregnancy. In all countries, inconsistencies between NMCPs and RH programmes on the timing or dose of IPTp-SP were documented, as was the mechanism for providing LLINs. Inconsistencies also were found in training documents from NMCPs and RH programmes in a given country. Outdated, inconsistent guidelines have the potential to cause confusion and lead to incorrect practices among health workers who implement MIP programmes, contributing to low coverage of IPTp-SP and LLINs.

**Conclusions:**

MIP policies, guidelines and training materials are outdated and/or inconsistent in the countries assessed. Updating and ensuring consistency among national MIP documents is needed, along with re-orientation and supervision of health workers to accelerate implementation of the 2012 WHO Global Malaria Programme policy recommendations for IPTp-SP.

## Background

The consequences of malaria in pregnancy (MIP) are well documented and include increased burden of maternal anaemia and low birth weight neonates in areas of stable malaria transmission [1]. The World Health Organization’s (WHO) three-pronged approach to the prevention of MIP includes: 1) the promotion and distribution of long-lasting insecticidal nets (LLINs)^a^ to pregnant women [2]; 2) intermittent preventive treatment of MIP with sulphadoxine-pyrimethamine (IPTp-SP); and 3) prompt diagnosis and effective treatment of malaria cases and maternal anaemia, or where diagnosis is not possible, treatment of reported fever as malaria [3,4]. In October 2012, the WHO Malaria Policy Advisory Committee reviewed evidence on the effectiveness of IPTp-SP in light of growing resistance to SP for treatment of *Plasmodium falciparum* malaria infection, and determined that use of IPTp-SP is effective in reducing the consequences of MIP, even in areas with high levels of SP resistance. The current WHO recommendation, updated in 2012, states that in areas of moderate to high malaria transmission in Africa, IPTp-SP should be given as early as possible in the second trimester and at each scheduled antenatal care (ANC) visit thereafter, with SP doses provided at least one month apart [5]. This same document states that high dose folate (≥5 mg) should not be given concomitantly with IPTp-SP, and reiterates the WHO recommendations that pregnant women receive 30–60 mg of elemental iron and 0.4 mg of folic acid daily to reduce the risk of maternal anaemia and iron deficiency at term. In addition, it notes that women receiving daily cotrimoxazole (CTX) prophylaxis should not receive IPTp-SP due to an increased risk of side effects; furthermore, daily CTX provides prophylaxis against malaria [6].

The Roll Back Malaria Partnership set global targets of 100% coverage of two doses of IPTp-SP in areas of high transmission and 80% use of LLINs by populations at risk by 2010 [7]. The President’s Malaria Initiative (PMI) works with national governments to deliver these interventions, with the goal to cover at least 85% of populations at risk [8]. While many sub-Saharan African countries have made headway towards these targets, current data show that progress has been disappointingly slow [9]; in the five included countries, uptake of two doses of IPTp-SP ranges from 11.2%–31.8%, and LLIN use by pregnant women ranges from 18.6%–71.3% (Table 1).

**Table 1:** Data on IPTp2 uptake and LLIN use by pregnant women (data source in parentheses)

**Country**	**Year of MIP policy adoption**	**IPTP2 uptake**	**LLIN use by pregnant women**
Kenya	2001	15.1% (2008–2009 DHS)	49% (2008–2009 DHS)
		25.4% (2010 MIS)	41.1% (2010 MIS)
Mali	2003	11.2% (2006 DHS)	55% (2010 MICS)
		20% (2012-13 preliminary DHS)	75.2% (2012-13 preliminary DHS)
Mozambique	2006	34.3% (2011 DHS)	18.6% (2011 DHS)
Mainland Tanzania	2002	27.2% (2010 DHS)	56.9% (2010 DHS)
		31.8% (2011-12 AIS/MIS)	74.8% (2011-12 AIS/MIS)
Uganda	2000	26.7% (2011 DHS)	46.9% (2011 DHS)

*DHS* Demographic and Health Survey, *MIS* Malaria Indicator Survey, *MICS* Malaria Indicator Cluster Survey, *HMIS* Health Management Information system, *AIS* AIDS Indicator Survey.

Reasons for low uptake of IPTp-SP and insecticide-treated bed nets (ITNs) are well-described in the literature [10-12] and can be understood from the vantage points of the pregnant woman, the health care worker and the health care system [11]. Health workers deal with challenges including: absence of written guidelines in their facilities on timing and dose of IPTp-SP, existence of written guidelines that are contradictory; conflicting recommendations from colleagues and supervisors; and lack of effective training and consistent supervision [11]. Guidelines for administration of IPTp-SP in some countries restrict its use to certain timeframes in pregnancy (i.e., 20–24 weeks and 28–32 weeks of pregnancy), which can cause uncertainty for health workers about how to treat a woman who attends ANC outside of these intervals. These issues lead to many missed opportunities to offer routine MIP preventive services during ANC.

The revised guidance on IPTp issued by WHO in October 2012 is an effort to reduce confusion among providers and increase coverage. National malaria control programmes (NMCPs), in collaboration with reproductive health (RH) programmes, are taking this opportunity to revise policies and guidelines used by frontline health workers. The programmes must seize this occasion to ensure that all policies, guidelines, training, and supervision documents contain correct, consistent and complete recommendations and clinical information on MIP, and that they are understandable to all levels of health workers. To inform this process, a review of the content of national-level MIP documents was undertaken in five sub-Saharan Africa countries to: 1) ascertain whether documents correctly and completely reflect WHO guidance on IPTp-SP use (2004) and treatment (2010), and LLIN use (2007); and 2) determine if documents from malaria control and RH programmes are consistent with each other.

## Methods

A review of national-level documents was undertaken in Kenya, Mali, Mozambique, mainland Tanzania and Uganda during the period November 1, 2012 to February 28, 2013. These countries were selected as a convenience sample of PMI countries with high likelihood of obtaining the necessary documents, which were requested from implementing partners, ministries of health and donors. Specific documents requested for the review included: 1) malaria and RH policies (the country’s guiding rules or principles to achieve national targets for malaria and maternal/newborn health [MNH]); 2) malaria and RH guidelines (the country’s clinical directives to health workers about prevention, diagnosis and treatment of malaria during pregnancy); 3) national training curricula, manuals and job aids for existing health care providers (in-service education) and for medical, midwifery and nursing students in basic education programmes (pre-service education); 4) and supervision guidelines. A standard framework^b^ was developed to capture pertinent information from each country’s documents relative to the WHO/Regional Office for Africa strategy issued in 2004 for IPTp-SP [13]; WHO/Global Malaria Programme’s 2007 guidance for LLIN use [2-14]; and WHO’s 2010 guidelines for treatment of malaria [15]. Documents in English, French and Portuguese were reviewed and data extracted into the framework by PG, a maternal and newborn health clinician with field experience in MIP programmes. While none of the country documents were expected to conform to the WHO policy recommendations issued in October 2012 for timing and dose of IPTp-SP, the framework reviewed current IPTp-SP guidelines produced by malaria control and RH divisions, and their consistency across documents. The key MIP areas assessed included: IPTp-SP timing and number of doses; concomitant use of IPTp-SP and folic acid [5]; directly observed therapy (DOT); malaria prevention for HIV-positive women taking daily CTX; LLIN promotion; LLIN distribution; diagnosis of malaria; and treatment of uncomplicated and severe malaria during pregnancy. It should be noted that implementation of the policies and guidelines contained in these documents was not assessed.

Documents were considered to conflict with each other if contradictory guidance was given. Inconsistent documents were defined as those where guidance was worded differently with the potential to cause confusion among programme managers and providers. Incomplete documents were defined as those who make little or no mention of a pre-specified key MIP area, such as use of DOT.

## Results

A total of 28 documents from the five countries were obtained. These included policy documents and guidelines from malaria and RH divisions, pre-service and in-service training materials, and supervision. The specific documents obtained from each country are detailed in Table 2. WHO guidance from 2004 was not reflected consistently in these documents, and within countries inconsistencies between malaria and RH guidelines and training materials were found. In addition, clear guidance on management of uncomplicated and severe malaria in pregnant women was often lacking. Table 3 presents WHO guidance for key MIP areas, as well as a summary of results of key MIP areas.

**Table 2:** National-level documents reviewed by country

	**National malaria policy**	**National malaria guidelines**	**National RH policy**	**National RH guidelines**	**In-service training materials**	**Supervision materials**	**Pre-service curriculum**
Kenya*	X (2010 – 2011)	X (2010)	X (2007)		X (2011)	X (2011)	
Mali	X (not dated but around 2008)	X (2012)	X (2004)	X (2004)	X (2011)	X (2012)	
Mozambique	X (2011)	X (2011)	X (2011)	X (2011)	X (2012)	X (2011)	X (2010)
Mainland Tanzania**		X (2006)		X (2003)	X (2008)	X (2011)	X (2010)
Uganda	X (2009)	X (not dated)	X (2012)	X (2012)	X (2011)		

“X” = document reviewed; parentheses indicate date document was published.

*Kenya does not have RH guidelines specific to provision of IPTp-SP during ANC.

**Mainland Tanzania does not have a separate malaria policy document. A national health policy document exists, but there is no separate RH policy.

**Table 3:** WHO guidance for key MIP areas and summary of document review results

**MIP area**	**WHO guidance**	**Document review results**
IPTp timing and dose	**IPTp timing**: Pregnant women should receive IPTp as early as possible in the second trimester of pregnancy and at every scheduled ANC visit thereafter, up to the time of delivery, at least one month apart. [5]	In all countries there were either no specific guidelines stating SP dose and gestational age at which IPTp-SP should be administered, or there was conflicting or inconsistent guidance; some countries recommended the first dose of IPTp-SP at 16 weeks, others after quickening, and others at 20–24 weeks; two countries prohibited IPTp-SP administration before 20 weeks and after 36 weeks. Four countries recommended specific weeks of pregnancy for IPTp-SP (i.e., first dose at 20–24 weeks, and second dose at 28–32 weeks).
	**IPTp dose**: Three tablets SP (each tablet containing 500 mg/25 mg SP). [5]	
	(WHO guidance from 2004: IPTp timing: at least two doses of IPTp-SP after quickening at least one month apart) [13]	
Prevention and treatment of anaemia	**Prevention and treatment of anaemia**: Folic acid at a daily dose equal or above 5 mg should not be given together with SP as this counteracts its efficacy as an antimalarial. Daily iron and folic acid supplementation of 30–60 mg of elemental iron and 0.4 mg of folic acid, to reduce the risk of low birth weight infants, maternal anaemia and iron deficiency at term [15].	Two countries that recommended high dose (5 mg) folic acid daily during pregnancy recommended interrupting folic acid intake: one country recommended one week after taking IPTp-SP, the other two weeks after taking IPTp-SP.
DOT	IPTp should be administered by DOT [15].	One country’s malaria policy and RH guidelines on case management did not specify that IPTp-SP be given by DOT. In that country, the malaria guidelines for IPTp stated that a prescription must be given to the pregnant woman who should be proceed to the on-site medication depot where an “agent” administers the IPTp-SP by DOT. All other countries recommend DOT by the ANC provider.
Linkages to HIV: Prevention of MIP for HIV-positive women	IPTp-SP is contraindicated for HIV-positive pregnant women taking CTX [5].	One country’s documents were consistent and reflected WHO guidelines. Three countries’ documents either made no mention of use of IPTp-SP for HIV-positive pregnant women or provided unclear guidance or guidance that conflicts with that of WHO.
	(WHO guidance from 2004: HIV-positive pregnant women will benefit from three to four doses of IPTp-SP at least one month apart) [13]	
LLIN promotion and distribution	ITNs should be provided to women as early in the pregnancy as possible, at the ANC clinic or through other sources in the public or private sectors [12].	All countries had policy recommendations for the use of LLINs as early as possible in pregnancy, but none had clear guidelines about when and how women should obtain them during ANC.
	The WHO Global Malaria Programme recommends distribution of ITNs, more specifically LLINs, to achieve full coverage of populations at risk of malaria. The best opportunity for rapidly scaling up malaria prevention is free or highly subsidized distribution of LLINs through existing public health services (both routine and campaigns) [2].	
Diagnosis	Diagnosis of MIP with microscopy or rapid diagnostic tests (RDTs) is recommended whenever possible [15].	Three countries had conflicting, incomplete and/or inconsistent guidelines about whether diagnostic tests should be performed prior to providing treatment to pregnant women for clinical malaria.
Treatment	**Uncomplicated malaria**	Two countries had at least one document that gave correct guidelines for treatment per trimester and severity of disease. But all countries had documents with incomplete and/or inconsistent guidelines for treatment of malaria by trimester.
	*First Trimester:* Quinine plus clindamycin to be given for seven days (artesunate plus clindamycin for seven days is indicated if this treatment fails). An artemisinin-based combination therapy (ACT) is indicated only if this is the only treatment immediately available, or if treatment with seven-day quinine plus clindamycin fails, or if there is uncertainty about patient compliance with a seven-day treatment. Note: If clindamycin is unavailable or unaffordable, then quinine monotherapy should be given.	
	*Second and Third Trimester:* ACT known to be effective in the country/region or artesunate plus clindamycin to be given for seven days or quinine plus clindamycin to be given for seven days (with the exception of DHA-PPQ (dihydroartemisinin-piperaquine) for which there is insufficient information to use it as a first-line therapy in second and third trimesters of pregnancy).	
	**Severe malaria:**	
	Parenteral anti-malarials should be given to pregnant women with severe malaria in full doses without delay. Parenteral artesunate is preferred over quinine in the second and third trimesters, because quinine is associated with recurrent hypoglycaemia. In the first trimester, the risk of hypoglycaemia is lower and uncertainties over the safety of artemisinin derivatives are greater, thus the two drugs are considered equivalent [15].	

### IPTp timing and dose

Documents from all five countries demonstrated conflicting and/or inconsistent guidance about timing and dose of IPTp-SP, although all countries recommended at least monthly intervals between doses (Table 4). For example, in Kenya the first dose of IPTp-SP was recommended after quickening in the national malaria guidelines, at 16 weeks or after quickening in the supervision guidelines and one in-service training package, and at 16 weeks in another in-service training package. Only the malaria guidelines specified the correct dose of SP for IPTp. In Mali, malaria guidelines recommended two doses of IPTp-SP, the first at 16 weeks and the second at least one month later and before the ninth month of pregnancy, while RH guidelines stated that the first dose should be given at 24–28 weeks, and the second at 32–36 weeks. The training package and supervision guidelines recommended two doses between the fourth and eighth months of pregnancy. Mozambique’s malaria guidelines stipulated two doses of IPTp-SP after 20 weeks while RH guidelines recommended three doses after 20 weeks, quickening or auscultation of foetal heart tones. In mainland Tanzania, malaria guidelines and in-service and pre-service education packages recommended the first dose of IPTp-SP at 20–24 weeks and the second dose at 28–32 weeks, while the quality improvement tool for ANC recommended the first dose at 20 weeks or more but gave no information about subsequent doses. Uganda’s malaria guidelines recommended the first dose between the fourth and sixth months, and the second between the seventh and ninth months of pregnancy, while the RH policy stipulated the first dose be given between 24–28 weeks, and the second at less than 36 weeks.

**Table 4:** IPTp timing inconsistencies within countries*

	**National malaria policy**	**National malaria guidelines**	**National RH policy**	**National RH guidelines**	**In-service training materials**	**Supervision materials**	**Pre-service curricula**	**Weeks during which providers can give IPTp-SP (# weeks during which IPT-SP can be given)**
Kenya	***No guidance***	Each scheduled visit after quickening up to 40 weeks	***No guidance***	***No guidance***	Each scheduled visit after quickening up to 40 weeks	Each scheduled visit after quickening up to 40 weeks	Not available	16–40 weeks **(24 weeks)**
Mali	***No guidance***	Twice after 16 weeks; no upper limit	Combined with RH guidelines, thus: IPTp-SP at 24–28 and 32–36 weeks	IPTp-SP at 24–28 and 32–36 weeks	Two doses between the 4^th^ and 8^th^ months of pregnancy, up to 36 weeks	Give between 4^th^ and 8^th^ months, number of doses not mentioned	Not available	Ranges from 16–40 weeks **(24 weeks);** from 24–28 and 32–36 weeks **(8 weeks**); and between the 4^th^–8^th^ months **(16 weeks)**
Mozambique	***No guidance***	Two doses after 20 weeks	***No guidance***	Three doses after 20 weeks	Give after 20 weeks	Three doses after 20 weeks	Three doses after 20 weeks	Give between 20–40 weeks; **(20 weeks)**
Mainland Tanzania	***No guidance***	1^st^ dose 20–24 weeks, 2^nd^ 28–32 weeks	***No guidance***	***No guidance***	1^st^ dose 20–24 weeks, 2^nd^ 28–32 weeks	1^st^ dose 20–24 weeks, 2^nd^ 28–32 weeks	1^st^ dose 20 – 24 weeks, 2^nd^ 28 – 32 weeks	Give between 20–24 and 28–32 weeks (**8 weeks)**
Uganda	***No guidance***	1^st^ dose 4^th^–6^th^ month, 2^nd^ dose 7^th^–9^th^ months	1^st^ dose at 24–28 weeks; 2^nd^ before 32 weeks	***No guidance***	1^st^ dose at 16–24 weeks; 2^nd^ dose at 28–34 weeks	***No guidance***	Not available	Ranges from 4^th^–9^th^ months **(24 weeks)**; between 24–28 weeks and before 32 weeks **(8 weeks**); and between 16–24 and 28–34 weeks **(14 weeks)**

*All countries stipulate that IPTp-SP should be given at intervals of at least 4 weeks.

### Anaemia prevention

At the time of this review, two countries (Kenya and Mali) routinely used high-dose folic acid (5 mg daily) during pregnancy in conjunction with iron supplementation. The WHO 2004 recommendations for IPTp-SP did not specify doses for iron or folate during pregnancy, though WHO 1998 guidelines recommended 60 mg iron and .25 mg – 0.4 mg folate daily [16]. Thus the use of higher dose folic acid was inconsistent with the WHO guidelines at that time. In Mali, only the Focused Antenatal Care (FANC) Reference Manual recommended suspending folic acid for one week after IPTp-SP. Kenya previously recommended suspending 5 mg folic acid for two weeks after IPTp-SP administration; however, the policy has since changed to provide folic acid 0.4 mg daily during pregnancy, and folic acid is no longer suspended.

### DOT

All countries except Mali had national malaria and RH policies and guidelines specifying that IPTp-SP should be given by DOT. In all four of these countries, national pre-service and in-service training documents also directed that IPTp-SP should be provided by DOT. In Mali, national malaria and RH guidelines did not specify that IPTp-SP should be administered under DOT, however the national malaria guidelines on IPTp-SP and ITNs directed health workers to give prescriptions for IPTp-SP that women take to the medicine depot, where DOT is carried out. In contrast, the in-service training document and the guidelines for supervision in Mali specified that IPTp-SP DOT be provided directly by health workers in ANC clinics.

### Linkages to HIV: Prevention of MIP for HIV-positive pregnant women

Four countries had inconsistent or incomplete policies and clinical guidelines about use of IPTp-SP for HIV-positive women. In Mali, the national malaria guidelines correctly recommended three doses of IPTp-SP for HIV-positive women, but did not state that IPTp-SP should not be given if the woman is taking daily CTX prophylaxis. In Mozambique, the RH guidelines and MNH nursing curriculum stated that women on CTX *or* antiretrovirals should not receive IPTp-SP (antiretrovirals alone are not a contraindication to IPTp-SP). In mainland Tanzania, the FANC Quality Improvement Tool stated that women with CD4 counts <350 cells/mm^3^ should take daily CTX, and that this should be stopped to give three doses of IPTp-SP, which conflicts with WHO guidelines. Finally, in Uganda, only one of four documents provided guidance on use of IPTp-SP for HIV-positive women, but did not discuss withholding IPTp-SP for women on CTX prophylaxis.

### LLIN promotion/distribution

All country documents recommended counselling pregnant women on the use of LLINs as early as possible in pregnancy, some specifying at the first ANC visit, but no country provided consistent guidance about how nets should be distributed, or how women can obtain them. While the supervision guidelines in mainland Tanzania recommended that providers give vouchers to women to subsidize the purchase of bed nets, the process is not clearly described in policies and guidelines. The malaria guidelines from Mozambique, and most documents from Kenya, Mali and Uganda recommended distribution of LLINs free of charge at the first ANC visit, but no specifics are given on who should provide them. It is also possible that LLIN promotional materials existed in these countries and were not captured in the documents reviewed, thus net voucher programmes or distribution campaigns might be established and operated outside health facilities and somewhat independent of national programmes.

### Diagnosis of MIP

Three countries had guidelines that were conflicting, inconsistent and/or incomplete when compared with the other documents from the country and with those from WHO about the need for diagnostic confirmation of clinical malaria with microscopy or RDTs. For example, Kenya’s malaria guidelines, one in-service training package and the supervision documents recommended parasitological diagnosis for all pregnant women reporting fever prior to treatment, while the second in-service training package did not mention testing prior to treatment. In Mozambique, the RH policy did not mention diagnosis, while all other documents stated diagnostic confirmation is required prior to treatment. Uganda’s malaria policy and guidelines recommend routine microscopy, and not RDTs, but also recommended that any pregnant woman presenting with fever be treated for malaria, even if the blood smear is negative. On the other hand, the malaria policy and in-service training material stated that either microscopy or RDTs should be available and used in all facilities. All of Mali’s and mainland Tanzania’s documents recommended parasitological confirmation of malaria before beginning treatment.

### Treatment of MIP

While four of five countries had at least one document that described treatment, specific to trimester of pregnancy and severity of infection, including drugs and their doses, none of the countries reviewed had consistent or complete treatment guidelines across all documents (Table 5)^c^. Kenya’s national malaria guidelines provided information, consistent with WHO treatment guidelines, for drugs and their doses by trimester for uncomplicated and severe malaria, but the two in-service training packages and supervision document provided incomplete, albeit consistent, information. None of Mali’s treatment guidelines stated the drug or dose according to trimester of pregnancy or severity of infection. Mozambique’s guidelines from both malaria and RH programmes contained complete, consistent treatment information, but the two in-service training packages and the MNH standards gave incomplete information. Uganda’s in-service training packages are the most complete, while the others lack information on drugs by trimester and severity of infection. Often they refer to guidelines for treatment of adults which did not always address the issue of treatment by trimester. Malaria guidelines in all five of the countries recommended parenteral quinine for severe malaria in second and third trimesters, instead of the WHO-recommended first-line drug, parenteral artesunate.

**Table 5:** Summary of consistency and completeness of MIP guidance among country-level documents (numerator is number of documents in each country with information about the MIP element, denominator is number of documents reviewed in a given country)

**Country/MIP element**	**Kenya**	**Mali**	**Mozambique**	**Mainland Tanzania**	**Uganda**
**IPTp timing**	4/4 documents are consistent, in accordance with 2004 WHO/Africa Regional Office Guidelines	2/4 documents give consistent information on timing; 1/4 is in accordance with WHO	3/9 documents do not specify timing or number of doses; 6/6 documents that mention timing are not in accordance with WHO; 3/6 documents that mention number of doses recommend two doses of SP, and 3/6 recommend 3 doses	4/4 documents are consistent on timing, though not in accordance with WHO; 75% state to give two doses of IPTp, 25% do not specify number	No consistency among five documents: one does not specify timing; one specifies first dose at 16 weeks; one at 20–24 weeks; and two in 4^th^–6^th^ months
**IPTp dose (SP, 3 tablets, at least 2 doses one month between doses)**	1/4 documents specify correct dose; 1/4 recommend use of SP in certain geographic areas	4/4 documents state correct SP dose	3/9 documents state SP dose	4/4 documents state correct SP dose	3/5 documents state correct SP dose
**Prevention and treatment of anaemia**	3/4 recommend suspension of folic acid for 14 days after SP	1/4 recommends suspension of folic acid for one week after SP			
**DOT**	4/4 documents recommend DOT	4/4 documents recommend DOT	9/9 documents recommend DOT	4/4 documents recommend DOT	3/4 documents recommend DOT
**Linkage to HIV**	4/4 documents recommend not giving SP to HIV-positive women on CTX	1/4 documents recommends not giving SP to HIV-positive women on CTX; two do not mention HIV-positive pregnant women; one states that they should receive three doses of IPTp	7/9 documents do not specify management of HIV-positive women; two state that women on CTX or antiretrovirals should not receive IPTp-SP	1/4 documents recommend not giving SP to HIV-positive women on CTX; 2/4 do not mention HIV-positive pregnant women; 1/4 documents is unclear	3/4 documents do not mention HIV-positive pregnant women; one document states need for three doses of IPTp, but incomplete guidance about use of SP for women on CTX
**ITN counselling**	4/4 documents recommend counselling	4/4 documents recommend counselling	6/9 documents recommend counselling	4/4 documents recommend counselling	4/4 documents recommend counselling
**ITN distribution to pregnant women during ANC**	2/4 documents specify distribution	3/4 documents specify distribution	3/9 documents specify distribution	1/4 documents specify distribution	1/4 documents specifies distribution
**Diagnostic testing**	3/4 documents recommend testing, 1/4 do not mention testing	4/4 documents recommend testing	8/9 documents recommend testing	4/4 documents recommend testing	3/4 documents recommend testing (only RH policy does not mention); one document recommending testing also states that any pregnant woman with fever should be treated for malaria even in the presence of a negative blood smear
**Treatment of uncomplicated malaria**	4/4 documents specify drug to be used; 1/4 documents specifies doses of drugs; 4/4 documents specify drugs by trimester; a; the document that mentions drugs and doses follow WHO treatment guidelines	4/4 documents specify drugs but no document gives doses, while 2/4 documents mention treatment by trimesters; given the incomplete information no document follows WHO treatment guidelines	6/6 documents specify drugs; 4/6 give dose and information by trimester; 2/6 documents are in accordance with WHO treatment guidelines	4/4 documents specify drugs; 2/4 documents state doses and 4/4 mention treatment by trimester; one document is consistent with WHO treatment guidelines	3/3 documents specify drugs, 2/3 mention doses, and 3/3 specify treatment by trimester; 1/3 documents is consistent with WHO guidelines
**Treatment of severe malaria**	4/4 documents specify drug to be used; 2/4 documents specify doses of drugs; 1/4 documents specifies trimesters. One document follows WHO treatment guidelines.	3/4 documents specify drugs to be used, and 2/4 state the dose, while one refers to treatment by trimester. One document follows WHO treatment guidelines.	6/6 documents specify drugs, 4/6 give doses and 5/6 specify trimesters. 3/6 documents follow WHO treatment guidelines.	4/4 documents specify drugs and doses; 3/4 specify treatment by trimester; no document is consistent with WHO treatment guidelines.	2/3 documents specify drugs, 1/3 gives doses, and 1/3 specifies treatment by trimester. No document is consistent with WHO treatment guidelines.

## Discussion and recommendations

This is the first formal review of national policies, guidelines, training and supervision materials for prevention and treatment of MIP across malaria control and RH programmes in Kenya, Mali, Mozambique, mainland Tanzania and Uganda. Important conflicts, inconsistencies and omissions were identified across all documents reviewed, which may contribute to health worker confusion and ultimately to challenges in scaling up these lifesaving interventions.

### IPTp timing, dose and DOT

Policy and guideline documents often provided conflicting and inconsistent information about use of IPTp-SP and restrictions on its timing. Four countries had out-of-date guidelines in terms of when IPTp-SP can be given, limiting opportunities to provide it, and contributing to low IPTp-SP coverage rates. Health care workers trained using these guidelines are placed in a position of not knowing whether to provide IPTp-SP between 16–24 weeks, as well as after 32 weeks, potentially shortening the window of opportunity to between 24–32 weeks. Since SP must be given no more often than monthly, few women in Mali’s system are likely to receive two doses of IPTp-SP at no more than monthly intervals during their pregnancies.

One challenge to implementation of the updated WHO recommendation for IPTp-SP is reassuring health workers that IPTp-SP can be given safely after completion of the first trimester, starting at 13 weeks of pregnancy, and up to the time of delivery, as long as doses are at least one month apart. Many guidelines recommend waiting until at least 20 weeks of pregnancy before giving the first dose of IPTp-SP. Countries should consider revising their policies and guidelines to recommend that all pregnant women receive their first dose of IPTp-SP starting at 13 weeks of pregnancy. All documents should utilize weeks, not months, of pregnancy to describe timing of IPTp-SP as they may not be understood in the same way. Country guidelines currently use terms such as “16 weeks or after quickening,” but because quickening may occur much later than 16 weeks, particularly in primigravid women, this can be a source of additional confusion. Provision of care will improve if health workers clearly understand in which specific week of pregnancy the first dose of IPTp-SP should be given. In addition, many guidelines recommend avoiding IPTp-SP in the last four weeks of pregnancy. The earliest WHO recommendations for IPTp recommended withholding IPTp during the last four weeks of pregnancy due to a theoretical risk that severe neonatal jaundice may be increased if sulfa drugs are provided near the time of delivery; however, this was unfounded, and the recommendation to withhold IPTp late in pregnancy was removed from the guidelines in 2004 [17]. Country policies should be updated to reflect these recommendations. In addition, ANC cards and registers may need to be updated so that health workers can easily document and monitor use of IPTp-SP.

While malaria control programmes provide technical guidance, MIP prevention activities are implemented by RH divisions through ANC. Failure to provide correct, consistent and complete MIP guidance in malaria and RH documents can lead to formulation of training and supervision documents and job aids that contain incomplete and/or conflicting information, resulting in confusion among ANC providers as to when IPTp-SP may be administered safely [10]. Thus pregnant women either may not receive services to prevent malaria, or services may be provided incorrectly. Conflicts and inconsistencies between RH and NMCP documents are difficult to prevent. Programmes update their documents at different intervals, based on programme-specific needs. Improving uptake of MIP interventions requires continued or renewed leadership from RH programmes, with technical support from NMCP and HIV control programmes. Until all of these programmes collaborate to formulate policies and guidelines for MIP prevention and treatment, discordant documents will continue to be issued within countries.

### Anaemia prevention

Since 1998 WHO has recommended daily doses of 0.4 mg of folic acid, which can be provided concurrently with SP, without compromising the efficacy of IPTp-SP. Interruptions in folic acid use can cause confusion for women about when to resume the folic acid, or may simply result in providers not prescribing IPTp-SP as they do not wish to disrupt folate administration. Countries should ensure as soon as feasible that the lower 0.4 mg dose of folic acid is provided to pregnant women. The change in folic acid dose and provision will require additional training and formulation of clear guidelines to health workers who are accustomed to counselling women to stop folic acid intake for periods of up to two weeks after receiving IPTp-SP.

### Linkages to HIV: Prevention of MIP for HIV-positive pregnant women

Guidelines from national HIV/AIDS control programmes were not reviewed, but across the five countries there are conflicting, inconsistent and/or incomplete guidelines from malaria control and RH programmes regarding malaria prevention for HIV-positive pregnant women and the contraindication to co-administration of CTX and IPTp-SP. Particularly, in light of updated WHO recommendations for prevention of mother-to-child transmission of HIV [18], all documents relating to malaria, RH and HIV should be updated to ensure correct and consistent guidelines about use of IPTp-SP for HIV-positive women; providers should be aware that while women taking CTX should not receive IPTp-SP, they still need to be encouraged to use LLINs. HIV-positive women not taking CTX should follow the same schedule of IPTp-SP use as HIV-negative women, since the updated WHO policy document recommends frequent dosing for all women. Revision of these documents should include representatives from the NMCP, RH programme, and HIV control programme so that prevention and treatment of MIP for HIV-positive pregnant women is clear for all health workers.

### Promotion and distribution of LLINs

Although most countries’ malaria and RH policies and guidelines recommend use of LLINs as early as possible in pregnancy, ideally at the first ANC visit, there is little or no information about when and how pregnant women are to obtain them. While some countries provide vouchers to women to subsidize their purchase, the process is not clearly described in policies and guidelines. In countries where women must purchase LLINs without vouchers, health workers need to know how to correctly inform women about when and where to obtain them. LLIN procurement and access to LLINs for pregnant women are particularly critical given the vulnerability of pregnant women and the fact that this is the only method of prevention available to pregnant women in the first trimester. Countries should make consistent recommendations about counselling of women on use of LLINs, as well as how nets are distributed. Ideally, LLINs should be given to all women, free of charge, at the first ANC visit, and this should be documented on the ANC card and register. Each country should clearly describe the mechanism for distribution, i.e. who in the ANC clinic is responsible for ensuring that the woman receives the net, or if vouchers are used, who provides the voucher and what instructions should be given about where and how to redeem them. In addition, countries should ensure that effective procurement and management practices prioritize adequate stocks of LLINs at ANC to give pregnant women consistent access and greater opportunities for coverage.

### Diagnosis and treatment

WHO recommends parasitological diagnosis whenever possible, yet many country guidelines are not correct, consistent or complete in recommending diagnosis of MIP with RDTs or microscopy prior to treatment. Treating all pregnant women with fever for presumed malaria can lead to over-treatment, exposure of the foetus to unnecessary medications and misdiagnosis of other causes of febrile illness. Although all country guidelines indicated the drugs to be used, many did not specify drug doses, nor did they state which drugs to give according to trimester of pregnancy or severity of infection. All clinical guidelines, educational materials and pertinent job aids should stress the need for parasitological diagnosis of MIP prior to treatment whenever possible. Clear algorithms—complete with correct drugs, doses, timing by trimester and by severity of infection—should be developed and used consistently in all documents and educational materials. User-friendly job aids that reflect these algorithms should be developed and distributed widely.

### Recommendations to achieve MIP programme goals

Most of the concerns mentioned above could be addressed by establishing a national technical working group (TWG) for MIP, comprised of leaders from the divisions of malaria, RH and HIV. Other key members should represent the departments responsible for laboratories/diagnostics, medical stores, pre-service and in-service education, health management information and monitoring/evaluation. The importance of TWGs across countries is well documented [19,20]. They provide an important mechanism not only to harmonize efforts, but also foster partnerships and create a working environment to coordinate and support programme implementation.

The TWG should identify and address gaps in the health system that are precluding uptake of IPTp-SP and LLINs in ANC clinics. Ideally, all elements of ANC should be scrutinized to ensure that women receive the complete package of services along with LLINs and IPTp-SP in an integrated manner. The TWG should engage all programmes linked with MNH to formulate a comprehensive checklist of services that every ANC clinic is responsible for providing and documenting. Ideally, RH programmes should lead the implementation of all interventions, including MIP, that ensure healthy outcomes for pregnant women and their newborns throughout the continuum of care, with technical oversight for MIP from the malaria control programme. It is important that NMCP inform RH colleagues of new or pending changes in malaria policies and guidelines so that RH documents reflect these in a timely manner. Policies and guidance documents for all ANC services, including MIP prevention and treatment, should be formulated jointly by NMCP, RH and related programmes in each country, so that inconsistencies can be averted. On a global level, WHO can ensure collaboration between the Department of Maternal, Newborn, Child and Adolescent Health and the Global Malaria Programme as new ANC guidelines are developed.

### Limitations

This review has some limitations. Not all documents requested from each country were available, and formal measurement was not done of how these national level documents affect implementation of MIP programmes. In addition, the convenience nature of our sample restricted the review to countries where at least some formal policy documents were reasonably available. As a result, the MIP policy situation in these five countries may be better than is typical across the rest of the malaria-endemic countries. As noted, information specific to MIP prevention or management may exist in documents outside those produced by the NMCP and RH programmes (such as guidelines for programmes implemented to prevent mother-to-child transmission of HIV), which would not have been considered in this review.

## Conclusion

The October 2012 WHO policy recommendation for IPTp-SP affords countries the opportunity to review their national-level policies and guidelines for malaria and RH and to update all national documents relating to prevention and treatment of MIP according to WHO guidelines. The WHO recommendation is also an opportunity for national malaria, RH and HIV programmes to collaborate in the revision, formulation and dissemination of correct, consistent and complete guidelines on MIP for all levels of health workers in all settings, and in ensuring that MIP becomes and remains a core component of integrated MNH services. To ensure rapid change and sustainable results, close collaboration between NMCP and RH, with RH taking ownership of MIP through ANC services, is a necessary ingredient to overcome inconsistencies in guidance documents. Scale-up of effective MIP services will result in attainment of international targets for uptake of IPTp-SP and LLINs, and, ultimately, in improved maternal and newborn outcomes.

## Endnotes

^a^This report refers to: 1) insecticide-treated nets (ITNs), which are the conventionally treated nets that have been dipped in insecticide and require retreatment after three washes or after one year of use; and 2) long-lasting insecticidal nets (LLINs), which are made with factory-treated netting material that incorporates insecticide within, or bound around, the fibres. These nets must retain their effectiveness without retreatment for at least 20 washes and three years of recommended use in the field. In 2007, WHO’s guidance to NMCPs and partners shifted to use of LLINs. Consistent with WHO’s guidance, the term “LLIN” is used in the recommendations of this report. The term “ITN” is used in this report where data and resources cited specify ITNs.

^b^ The framework mentioned is available at: http://www.mchip.net/node/1813.

^c^ Guidelines are considered complete if they contain drug names, routes, doses and duration, as well as use by trimester of pregnancy and severity of infection.

## Abbreviations

ACT: Artemisinin-based combination therapy; ANC: Antenatal care; CTX: Cotrimoxazole; DOT: Directly observed therapy; FANC: Focused antenatal care; HIV/AIDS: Human immunodeficiency virus/acquired immunodeficiency syndrome; IPTp: Intermittent preventive treatment in pregnancy; IPTp-SP: Intermittent preventive treatment in pregnancy with sulphadoxine-pyrimethamine; ITN: Insecticide-treated bed net; LLIN: Long-lasting insecticide-treated bed net; MIP: Malaria in pregnancy; MNH: Maternal and newborn health; NMCP: National Malaria Control Programme; RDT: Rapid diagnostic test; RH: Reproductive health; SP: Sulphadoxine-pyrimethamine; TWG: Technical working group; WHO: World Health Organization.

## Competing interests

The authors declare that they have no competing interests.

## Authors’ contributions

PG extracted the data. AD, PG, JG, and ER, contributed to the analysis of data. The draft was written by PG. MJH, JG, ER, AD, EE, SY and ZA made substantive contributions to structure and content. All authors have read and approved the final manuscript. PG is the corresponding author.
